# Supplementary material for: Evidence That DDR1 Promotes Oligodendrocyte Differentiation during Development and Myelin Repair after Injury
Source: Int J Mol Sci. 2023 Jun 19;24(12):10318. doi: 10.3390/ijms241210318 (PMC10299687; doi:10.3390/ijms241210318)
Supplement: Supplementary file 1 [file ijms-24-10318-s001.zip › ijms-2420564-supplementary.pdf]

Suppelemantary materials

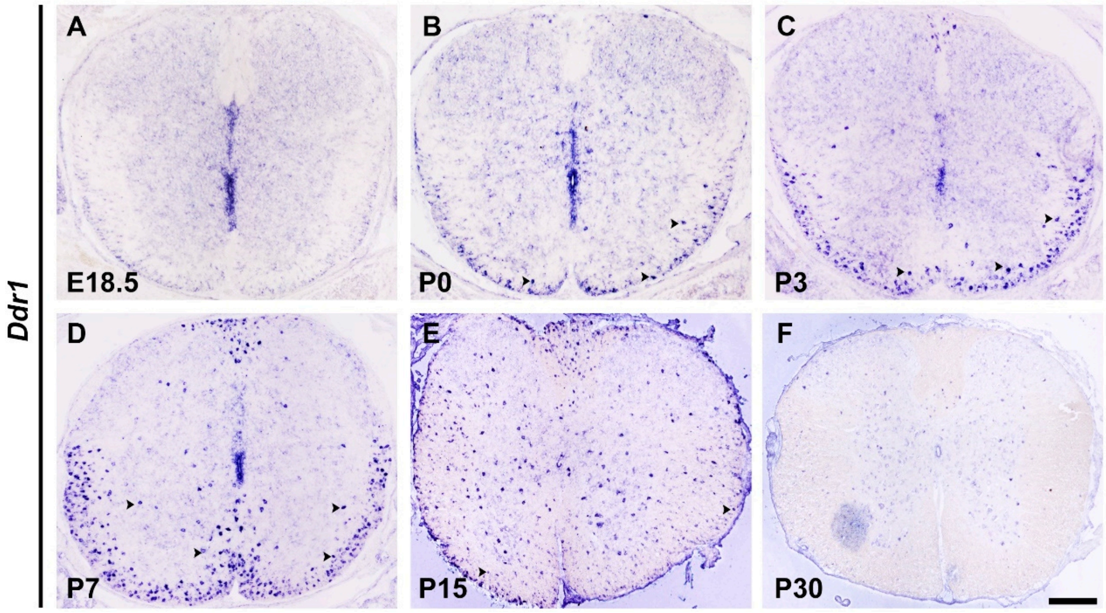

Figure S1. Developmental expression pattern of *Ddr1* in the spinal cord. **(A-F)** *Ddr1* ISH in mouse spinal cord from E18.5 to P30. Scale bar represents 25 μm. Black arrowheads highlight *Ddr1* positive cells.

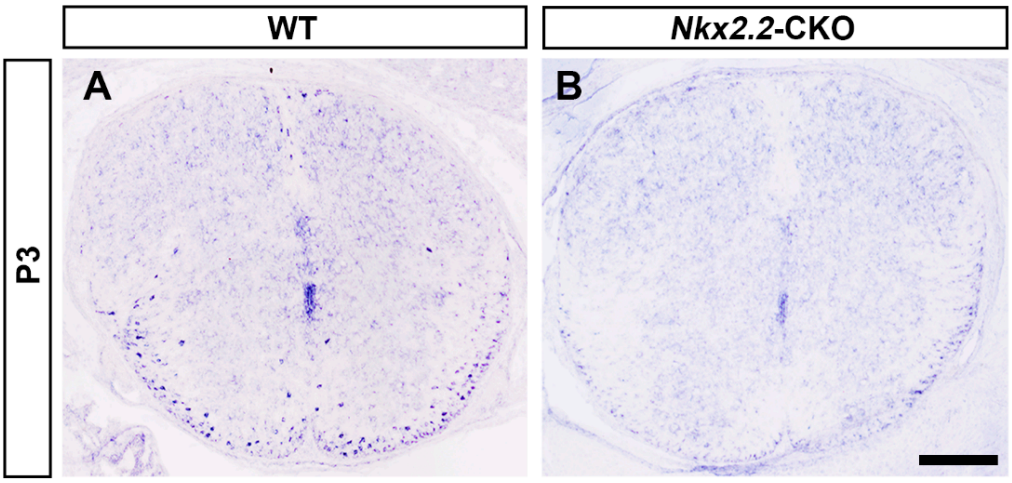

Figure S2. Expression of *Ddr1* in *Nkx2.2*-cKO mutant spinal cords is dramatically reduced at P3 stages. Scale bar represents 100 μm.
